# Coexpression and interaction of CXCL10 and CD26 in mesenchymal cells by synergising inflammatory cytokines: CXCL8 and CXCL10 are discriminative markers for autoimmune arthropathies

**DOI:** 10.1186/ar1997

**Published:** 2006-07-17

**Authors:** Paul Proost, Sofie Struyf, Tamara Loos, Mieke Gouwy, Evemie Schutyser, René Conings, Isabelle Ronsse, Marc Parmentier, Bernard Grillet, Ghislain Opdenakker, Jan Balzarini, Jo Van Damme

**Affiliations:** 1 Laboratory of Molecular Immunology, Rega Institute for Medical Research, KU Leuven, Leuven, Belgium; 2 IRIBHN, Université Libre de Bruxelles, Campus Erasme, Brussels, Belgium; 3 Laboratory of Immunobiology, Rega Institute for Medical Research, KU Leuven, Leuven, Belgium; 4 Ziekenhuis Zeeuws-Vlaanderen, Terneuzen, The Netherlands; 5 Laboratory of Virology and Chemotherapy, Rega Institute for Medical Research, KU Leuven, Leuven, Belgium

## Abstract

Leukocyte infiltration during acute and chronic inflammation is regulated by exogenous and endogenous factors, including cytokines, chemokines and proteases. Stimulation of fibroblasts and human microvascular endothelial cells with the inflammatory cytokines interleukin-1β (IL-1β) or tumour necrosis factor alpha (TNF-α) combined with either interferon-α (IFN-α), IFN-β or IFN-γ resulted in a synergistic induction of the CXC chemokine CXCL10, but not of the neutrophil chemoattractant CXCL8. In contrast, simultaneous stimulation with different IFN types did not result in a synergistic CXCL10 protein induction. Purification of natural CXCL10 from the conditioned medium of fibroblasts led to the isolation of CD26/dipeptidyl peptidase IV-processed CXCL10 missing two NH_2_-terminal residues. In contrast to intact CXCL10, NH_2_-terminally truncated CXCL10(3–77) did not induce extracellular signal-regulated kinase 1/2 or Akt/protein kinase B phosphorylation in CXC chemokine receptor 3-transfected cells. Together with the expression of CXCL10, the expression of membrane-bound CD26/dipeptidyl peptidase IV was also upregulated in fibroblasts by IFN-γ, by IFN-γ plus IL-1β or by IFN-γ plus TNF-α. This provides a negative feedback for CXCL10-dependent chemotaxis of activated T cells and natural killer cells. Since TNF-α and IL-1β are implicated in arthritis, synovial concentrations of CXCL8 and CXCL10 were compared in patients suffering from crystal arthritis, ankylosing spondylitis, psoriatic arthritis and rheumatoid arthritis. All three groups of autoimmune arthritis patients (ankylosing spondylitis, psoriatic arthritis and rheumatoid arthritis) had significantly increased synovial CXCL10 levels compared with crystal arthritis patients. In contrast, compared with crystal arthritis, only rheumatoid arthritis patients, and not ankylosing spondylitis or psoriatic arthritis patients, had significantly higher synovial CXCL8 concentrations. Synovial concentrations of the neutrophil chemoattractant CXCL8 may therefore be useful to discriminate between autoimmune arthritis types.

## Introduction

Chemokines form a family of chemotactic cytokines that are classified as CXC or CC according to the positioning of NH_2_-terminal cysteines in their primary protein structure [1,2]. CXC ligands (CXCL) can be further distinguished based on their receptor (CXCR) usage. For example, CXCL8 (IL-8) recognises CXCR1 and CXCR2 and selectively chemoattracts neutrophilic granulocytes, whereas CXCR3 ligands (e.g. CXCL10 – interferon (IFN)-inducible protein-10/IP-10) are specific attractants for lymphocytes and natural killer cells [3,4]. Fibroblasts and capillary endothelial cells are important cellular sources of CXCL8, produced in response to various stimuli including exogenous microbial products and proinflammatory cytokines such as IL-1β and tumour necrosis factor alpha (TNF-α) [5,6].

Some subsets of chemokines, such as the CXCR3 ligands, were discovered as proteins specifically induced by IFNs in selected cell types, such as astrocytes and keratinocytes [7-10]. In addition, some Toll-like receptor ligands (e.g. double-stranded RNA) stimulate the production of these CXCR3 ligands in leukocytes and fibroblasts [11,12]. Moreover, such microbial products (e.g. lipopolysaccharide (LPS)) synergise with IFN-γ to drastically enhance the production of CXCL10 by fibroblasts and to inhibit IFN-γ-induced CXCR3 ligand production in peripheral blood mononuclear cells [11,12].

Synergy between TNF-α and IFN-γ to induce CXCL10 has previously been reported for several cell types, including leukocytes, epithelial cells, endothelial cells and fibroblasts [13-16]. In endothelial cells and fibroblasts, however, most other cytokine combinations have not been evaluated for induction of CXCR3 ligands [17]. Simultaneously, these inflammatory cytokines induce the expression of matrix degrading metalloproteases (e.g. gelatinase, collagenase) in these cell types [18,19].

In addition, IL-1β and IFN-γ have been reported to stimulate the expression of other chemokine processing proteases such as CD26/dipeptidyl peptidaseIV (DPP IV) (EC 3.4.14.5) in fibroblasts, whereas for TNF-α the regulation of CD26/DPP IV expression in fibroblasts is rather controversial [20,21]. Moreover, nothing is known about the regulation of the expression of such enzymes when cytokines act simultaneously. CXCR3 ligands are nevertheless good substrates for CD26/DPP IV, which inactivates the CXCR3 ligands as chemoattractants [22].

Cytokines and proteases, derived from synovial fibroblasts, endothelial cells or leukocytes, are key players of the immune response and strongly interact in inflammatory disorders such as autoimmune arthritis. IL-1β and TNF-α are clearly implicated in the pathogenesis of rheumatoid arthritis (RA) since blockage of their activities by antibodies or receptor antagonists is beneficial for patient treatment [23,24]. CXCR3 and CD26/DPP IV are highly expressed on activated T helper type 1 (Th1) lymphocytes, which compose the majority of infiltrating T cells in the synovial cavity of RA patients [25,26]. In addition, synovial fibroblasts also strongly express CD26/DPP IV [27]. Moreover, patients suffering from RA showed reduced serum, but not synovial fluid, CD26/DPP IV activity compared with osteoarthritis patients [25].

In order to obtain more insight into CXCL10 processing and the role of CXCL10 and CD26/DPP IV in various rheumatic diseases, including psoriatic arthritis (PsA), ankylosing spondylitis (AS) and RA, the synergistic interaction between cytokines to regulate CXCL10 and CD26/DPP IV expression in fibroblasts and endothelial cells was investigated. The regulated production of the lymphocyte chemoattractant CXCL10 was compared with the induction of the neutrophil chemotactic protein CXCL8, and synovial concentrations of both chemokines were compared in RA, PsA, AS and crystal-induced arthritis (CA).

## Materials and methods

### Reagents

Recombinant human IL-1β, TNF-α, IFN-γ and CXCL10 were purchased from PeproTech (Rocky Hill, NJ, USA). Recombinant CXCL10(3–77) was generated by cleaving intact CXCL10 with CD26/DPP IV as previously described [22], and was purified to homogeneity by reverse-phase (RP)-HPLC (C8 Aquapore RP-300 column, 1 × 50 mm; Applied Biosystems, Foster City, CA, USA). Recombinant IFN-α (Roferon A) was obtained from Hoffman-La Roche (Nutley, NJ, USA) and IFN-β (Avonex) was bought from Biogen (Cambridge, MA, USA). Natural human CXCL8 was purified from conditioned medium of leukocytes as previously described [28]. The Limulus amebocyte lysate assay (Cambrex Bio Science, Verviers, Belgium) was used for measuring endotoxin levels, which were <2 pg LPS per 10^6 ^U IFN-α or IFN-β, <1 pg LPS per μg IFN-γ or TNF-α and <2 pg LPS per 10^4 ^U IL-1β.

### Cell cultures and induction experiments

Human diploid skin/muscle-derived fibroblasts (E1SM) were grown in MEM containing 10% (v/v) foetal bovine serum (FBS) (Cambrex Bio Science) [12]. Fibroblast monolayers were grown to confluency in 24-well plates (1 ml/1.9 cm^2^, 3–10 days after subcultivation; ± 50,000 cells/cm^2^) and inducers were supplemented to 1 ml MEM containing 10% (v/v) FBS. Conditioned media were harvested after 72 hours. Human dermal neonatal microvascular endothelial cells from pooled donors (HMVEC; Cambrex Bio Science) were cultured in endothelial basal medium-2 containing endothelial growth medium EGM-2MV SingleQuots (Cambrex Bio Science). HMVEC were seeded in 48-well dishes and induced 5 days after subcultivation (± 10,000 cells/cm^2^) with cytokines in complete growth medium (0.5 ml/well) for 72 hours.

### Immunoassays

Levels of human CXCL8 and CXCL10 were quantified by specific sandwich ELISAs developed in our laboratory as previously described [22]. Briefly, 96-well plates (Maxisorp; Nunc-Immuno Plate, Roskilde, Denmark; and Greiner Bio-One, Kremsmuenster, Austria) were coated with goat polyclonal anti-human CXCL8 antibodies, which were generated in our laboratory [29], followed by blocking with PBS containing 0.1% casein and 0.05% Tween-20. The capture of human CXCL8 in test samples (cell culture supernatants or synovial fluids) was detected by mouse monoclonal anti-human CXCL8 antibody (R&D Systems, Abingdon, UK) and by a secondary antibody, peroxidase-conjugated anti-mouse IgG (Jackson ImmunoResearch Laboratories, West Grove, PA, USA). Peroxidase activity was quantified by measuring the conversion of 3,3',5,5'-tetramethylbenzidine (Sigma-Aldrich, St Louis, MO, USA) at 450 nm.

The sandwich ELISA for human CXCL10 consisted of mouse monoclonal anti-human CXCL10 (R&D Systems) as a coating antibody, biotinylated rabbit polyclonal anti-human CXCL10 (R&D Systems) as a capturing antibody and peroxidase-conjugated streptavidin (Jackson ImmunoResearch Laboratories) as a detecting antibody.

These ELISAs did not show cross-reactivity with any other chemokine or any used chemokine inducer. Human soluble CD26 was determined with a commercially available sandwich ELISA (Bender MedSystems, Vienna, Austria) that had a detection limit of 16 ng/ml CD26 protein.

### Purification and identification of natural chemokines

Confluent fibroblast cultures (80 culture flasks of 175 cm^2^) were induced by combined treatment with IFN-γ (20 ng/ml), LPS (5 μg/ml) and double-stranded RNA (10 μg/ml) for 96 hours to obtain maximal production of CXCL10 [11]. The conditioned medium (2 l) was first concentrated by adsorption to controlled pore glass (1/30 v/v CPG-10-350; Serva, Heidelberg, Germany) as previously described [28]. Chemokines were subsequently loaded onto a heparin Sepharose-CL-6B column (Amersham Biosciences, Roosendaal, The Netherlands) in 50 mM Tris (pH 7.4) containing 50 mM NaCl and were eluted in a NaCl gradient (50 mM to 2 M NaCl in 50 mM Tris, pH 7.4). Fractions containing CXCL10 immunoreactivity were dialysed against 50 mM formic acid (pH 4.0) and loaded onto a 1 ml MonoS (Amersham Biosciences) cation exchange chromatography column. Proteins were eluted from the cation exchanger in a NaCl gradient (0–1 M in 50 mM formic acid, pH 4.0) and loaded onto a C8 RP-HPLC column (2.1 × 220 mm Aquapore RP-300 column; Applied Biosystems) in 0.1% (v/v) trifluoroacetic acid. Chemokines were eluted from the column in an acetonitrile gradient (0–80 v/v% in 0.1% trifluoroacetic acid) and proteins were detected in the eluent at 214 nm.

From the RP-HPLC eluent, 0.7% was split to an electrospray ion trap mass spectrometer (Esquire LC; Bruker Daltonics, Bremen, Germany). Spectra were averaged over the chromatographic peaks detected at 214 nm, and the relative molecular mass (*M*_r_) of proteins was calculated with the Bruker deconvolution software. In addition, the NH_2_-terminal sequence of chemokines was determined by Edman degradation on a capillary protein sequencer (Procise 491cLC; Applied Biosystems).

### CD26/DPP IV activity assays

The DPP IV activity was detected with a substrate conversion assay [30]. Briefly, confluent fibroblast monolayers were washed with serum-free medium and treated with cytokines. After 48 hours, 200 μl conditioned medium was removed and incubated with GlyPro-*p*-nitroanilide (3 mM final concentration). Alternatively, cytokines were added to confluent fibroblast monolayers, and cells were washed with PBS after 96 hours and incubated with 200 μl PBS containing 3 mM GlyPro-*p*-nitroanilide. The increase of the UV absorption at 400 nm (OD_400_) caused by the DPP IV-catalysed proteolytic release of *p*-nitroanilide from GlyPro-*p*-nitroanilide was monitored at 37°C in a Spectramax microplate spectrophotometer (Molecular Devices, Sunnyvale, CA, USA). The OD_400 _values of the reaction mixtures before the addition of GlyPro-*p*-nitroanilide were subtracted from the obtained values to represent the real increase of OD_400 _values as a measurement of proteolytic activity.

### Fluorescence-activated cell sorting (FACS) analysis

Confluent fibroblast monolayers (in six-well plates, 9 cm^2^/well) were incubated with cytokines for 48 hours and were subsequently trypsinised. Cells were stained with anti-human CD26 antibody (BD Biosciences, Erembodegem, Belgium) in PBS containing 2% FBS. After two washing steps with PBS containing 2% FBS, the secondary antibody PE-conjugated goat anti-mouse Ig (BD Biosciences) was added to the cell suspension. Subsequently, the PE-stained fibroblasts were fixed in PBS containing 2% (v/v) formaldehyde and analysed on a BD FACSCalibur cytometer (BD Biosciences) using the CellQuest software (BD Biosciences), collecting 10,000 events/sample.

### Signal transduction assays

The Chinese hamster ovary (CHO) cell line transfected with CXCR3 was cultured in Ham's F-12 growth medium (Cambrex Bio Science) enriched with 10% FBS (Invitrogen), 400 μg/ml G418 and 1 mM sodium pyruvate [22]. Before stimulation, 0.5 × 10^6 ^cells (in 2 ml) were seeded in a six-well plate (9 cm^2^; Techno Plastic Products AG, Trasadingen, Switzerland) in Ham's F-12 medium supplemented with 10% FBS. After 24 hours, the growth medium was removed and the cells were cultured overnight in serum-free starvation medium. The starvation medium was subsequently removed and 900 μl Ham's F-12 medium supplemented with 0.5% FCS was added to each well. The cells were preincubated at 37°C for 15 minutes before stimulation with the test sample (diluted in 100 μl Ham's F-12 medium) for 5 minutes at 37°C. Signal transduction was stopped by chilling the cell culture plates on ice and adding ice-cold PBS. Afterwards, cells were washed twice with ice-cold PBS and cell lysis was performed in PBS containing 1 mM ethylenediamine tetraacetic acid, 0.5% Triton X-100, 5 mM NaF, 6 M urea, protease inhibitor cocktail for mammalian tissues and phosphatase inhibitor cocktails 1 and 2 (Sigma-Aldrich) (100 μl/well). After 10 minutes cells were scraped off, and the lysate was collected, was incubated for 45 minutes on ice and was clarified (10 min, 1200 × *g*).

The protein concentration in the supernatant was determined by the bicinchoninic acid protein assay (Pierce, Rockford, IL, USA). The amount of extracellular signal-regulated kinase 1/2 (ERK1/2) and protein kinase B/Akt phosphorylation in the supernatant (in picograms of phospho-ERK1/2 or phospho-Akt per milligram of total protein) was determined using specific ELISAs for phospho-ERK1 (T202/Y204), for phospho-ERK2 (T185/Y187) or for phospho-Akt (S473) (R&D Systems).

### Patients

Synovial fluids from patients with RA (*n *= 71), patients with AS (*n *= 18), patients with PsA (*n *= 14) or patients with CA (*n *= 23) were collected in dry tubes and centrifuged for 4 minutes at 1000 rpm. Aliquots were immediately frozen at -20°C until analysis. The RA patients fulfilled the revised American College of Rheumatology criteria. The AS patients were diagnosed according to the modified New York criteria. Arthritis in patients with psoriasis was defined as PsA. CA was diagnosed when either calcium pyrophosphate dihydrate or uric acid were detected in the synovial fluid by polarised light microscopy.

Informed consent was obtained from all patients and procedures followed the tenets of the Declaration of Helsinki. The Ethical Committee of the University of Leuven approved the study.

## Results

Synergistic induction of CXCL10 ligands in fibroblasts and endothelial cells by inflammatory cytokines.

The lymphocyte chemotactic CXCR3 ligands are known to be inducible by IFNs, whereas IL-1β and TNF-α are potent inducers of several other chemokines such as the main CXCR1 and CXCR2 ligand CXCL8. IL-1β, TNF-α and IFNs are often coproduced during inflammation. The ability of combinations of these cytokines to induce CXCL8 and CXCL10 in fibroblasts was therefore investigated.

**Figure 1:**
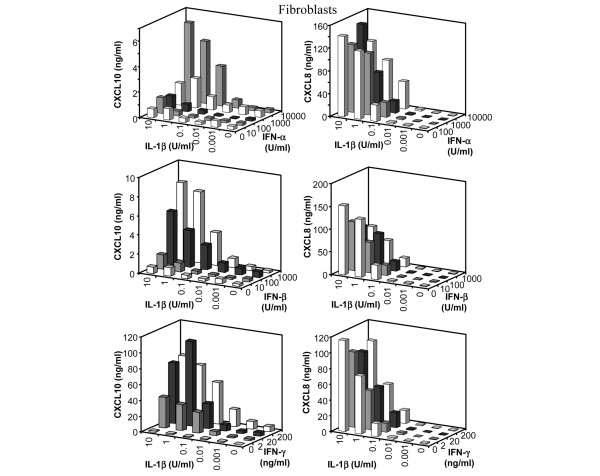
**CXCL8 and CXCL10 induction in fibroblasts by IL-1β and interferons**. Confluent fibroblast monolayers were incubated with IL-1β in combination with IFN-α, IFN-β or IFN-γ. Results represent the mean CXCL8 and CXCL10 protein concentration (ng/ml) measured in the culture supernatant (three or more independent experiments).

**Figure 2:**
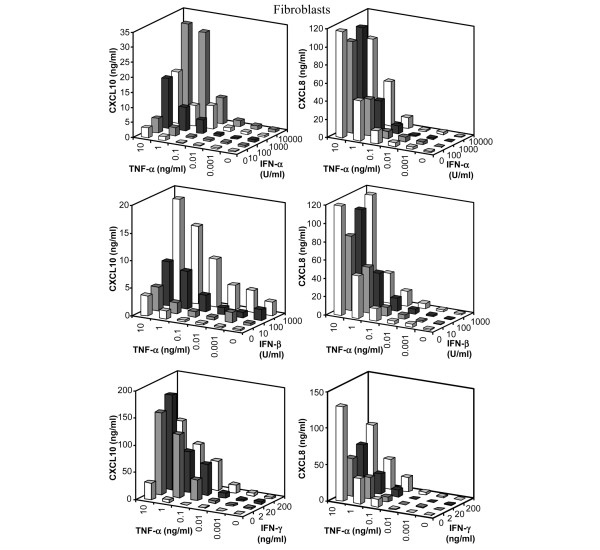
**CXCL8 and CXCL10 induction in fibroblasts by tumour necrosis factor alpha and interferons**. Confluent fibroblast monolayers were incubated with tumour necrosis factor alpha (TNF-α) in combination with IFN-α, IFN-β or IFN-γ. Results represent the mean CXCL8 and CXCL10 concentration (ng/ml) measured in the culture supernatant (three or more independent experiments).

**Figure 3:**
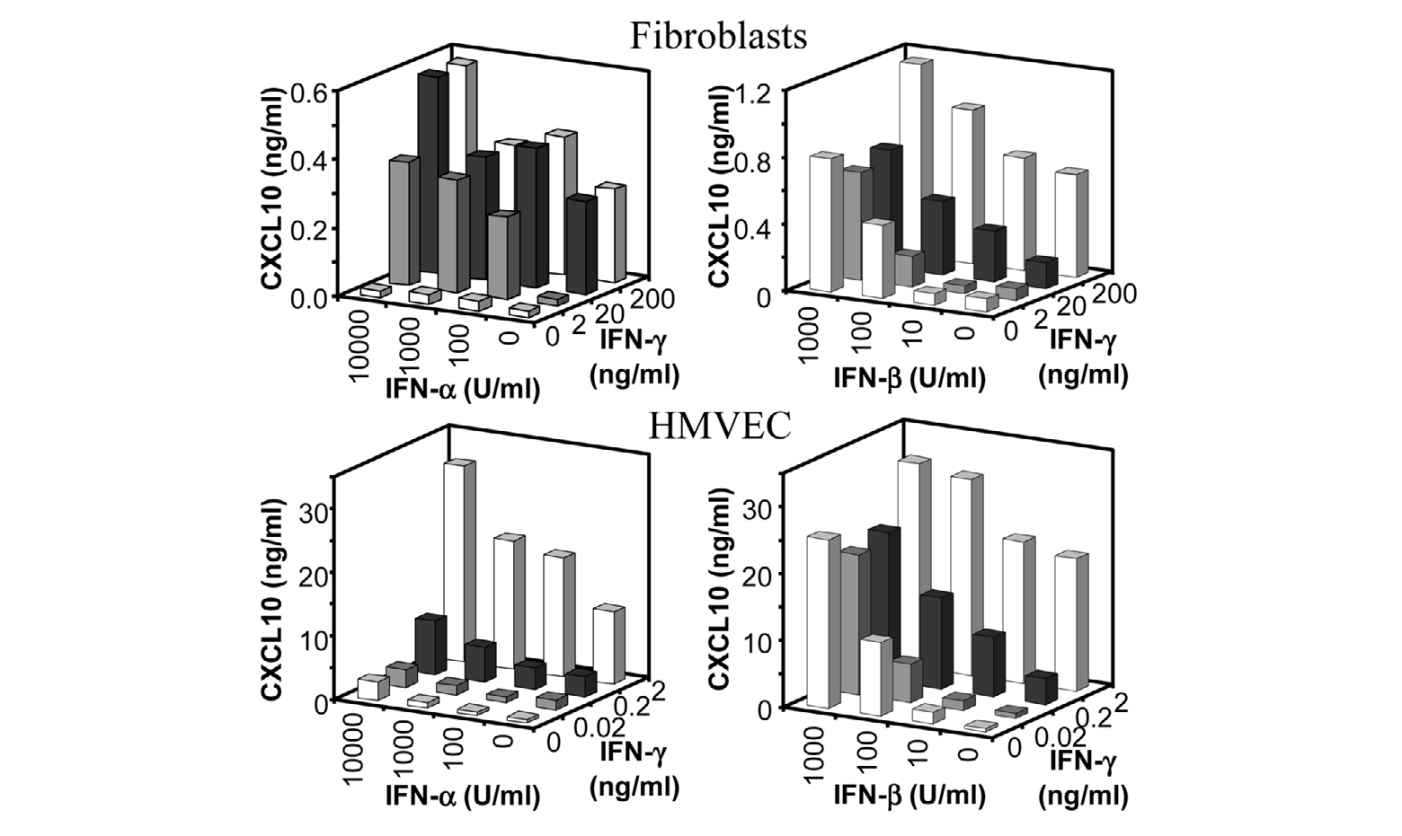
**CXCL10 induction by combinations of interferons in fibroblasts and human microvascular endothelial cells**. Monolayers of fibroblasts or human microvascular endothelial cells (HMVEC) were incubated with combinations of IFN-α or IFN-β and IFN-γ. Results represent the mean CXCL10 concentration (ng/ml) measured in the culture supernatant (three or more independent experiments).

Diploid fibroblasts were grown to confluency and were stimulated with IL-1β (0.001–10 U/ml) or TNF-α (0.001–10 ng/ml) in conditioned media in the presence of IFN-α (10–10,000 U/ml), IFN-β (10–1000 U/ml) or IFN-γ (2–200 ng/ml) for 72 hours. The culture medium was then analysed for CXCL10 production by specific ELISA. Although IL-1β and TNF-α as well as IFN-α, IFN-β or IFN-γ were rather weak inducers of CXCL10 (1–5 ng/ml) in fibroblasts as single agents, all combinations provided a dose-dependent synergistic induction yielding a 3-fold to 30-fold increase of CXCL10 production (5–150 ng/ml) (Figures 1 and 2, left panels). In particular, induction of fibroblasts with IL-1β or TNF-α together with IFN-γ (2–200 ng/ml) provided a strong synergistic effect (up to 50-fold increase above the additive effect for IL-1β and IFN-γ). Stimulation of fibroblasts with IFN-α plus IFN-γ or with IFN-β plus IFN-γ (Figure 3), however, only yielded a weak synergistic CXCL10 induction and the total CXCL10 production remained low (≤1 ng/ml). This indicates that the synergy with IFN-γ does not indirectly depend on the induction of IFN-β on the fibroblasts by IL-1β or TNF-α.

In addition, the production of CXCL8, the chemokine with the highest specific activity on neutrophilic granulocytes, was determined after stimulation of fibroblasts with IL-1β or TNF-α in the presence of IFN-α, IFN-β or IFN-γ (Figures 1 and 2, right panels). IL-1β (1 U/ml) and TNF-α (10 ng/ml) alone induced more than 100 ng/ml CXCL8. The presence of IFN-β or IFN-γ rather moderately and dose-dependently inhibited the production of CXCL8 in response to IL-1β. Finally, fibroblast treatment with single or combined IFN types did not result in CXCL8 production (data not shown). It can be concluded that IFNs in fibroblasts inhibit CXCL8 production, whereas IFNs in combination with IL-1β or TNF-α synergistically stimulate production of CXCL10.

**Figure 4:**
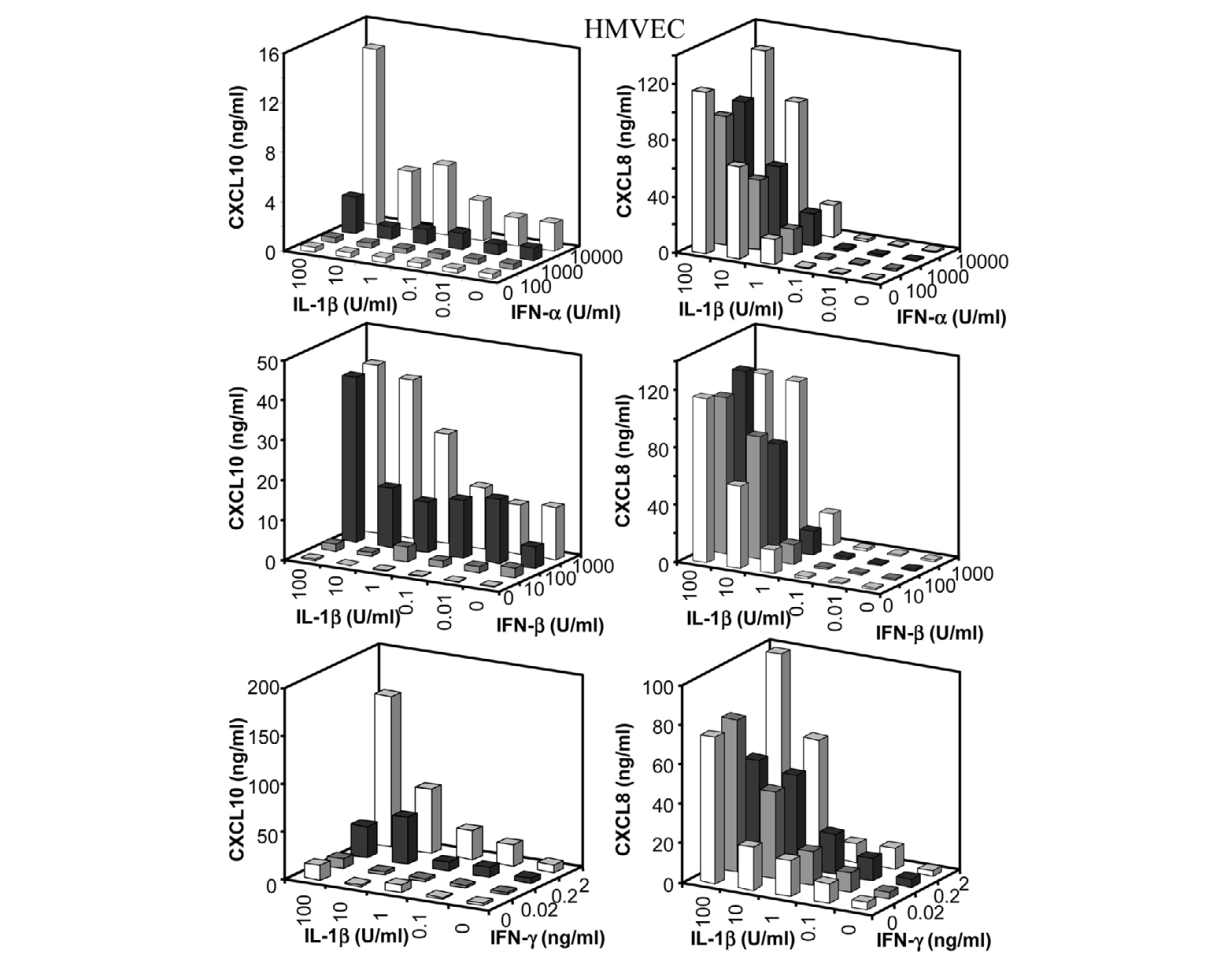
**CXCL8 and CXCL10 induction in human microvascular endothelial cells by IL-1β and interferons**. Human microvascular endothelial cells (HMVEC) were incubated with IL-1β in combination with IFN-α, IFN-β or IFN-γ. Results represent the mean CXCL8 and CXCL10 concentration (ng/ml) measured in the culture supernatant (three or more independent experiments).

**Figure 5:**
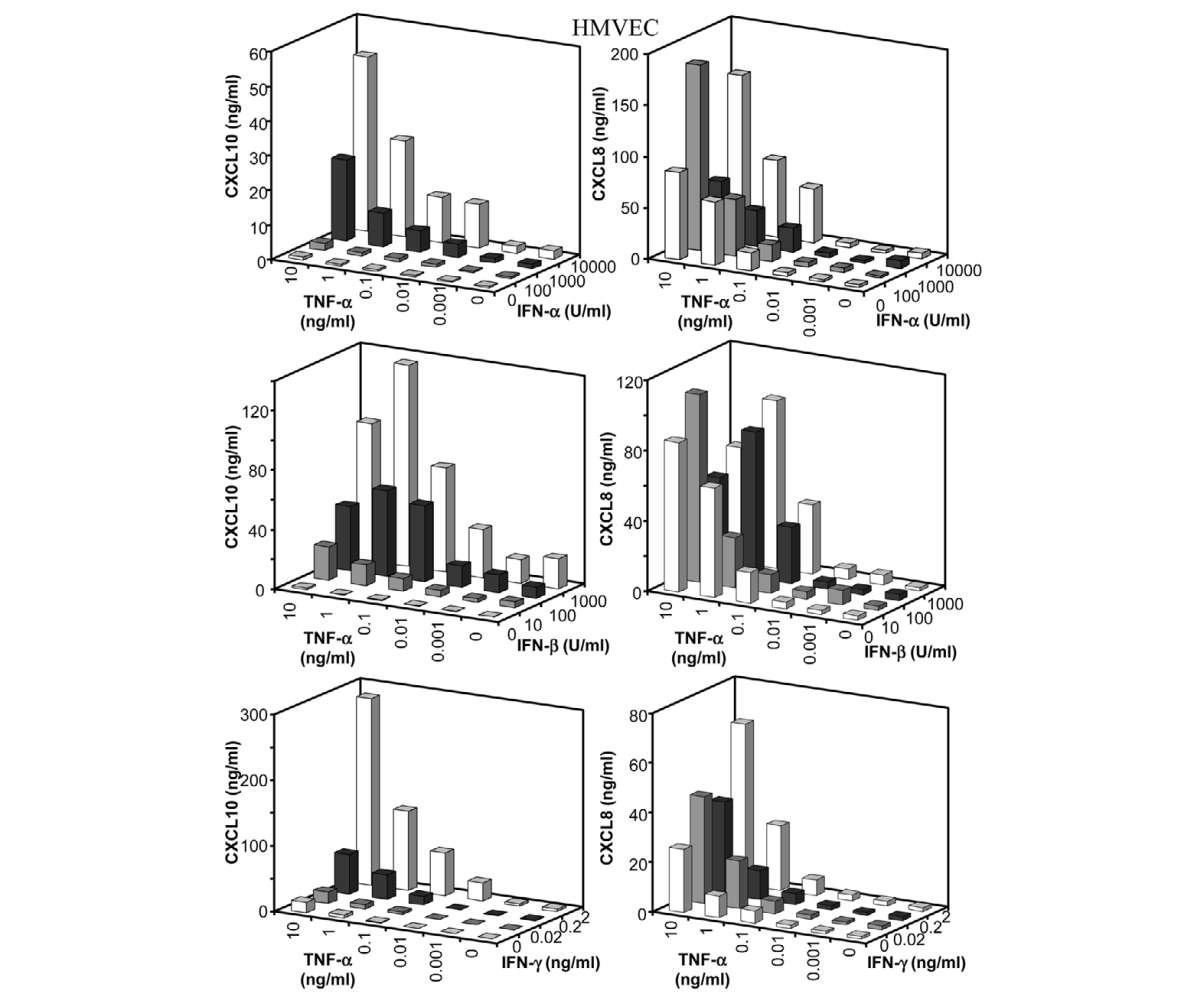
**CXCL8 and CXCL10 induction in HMVEC by tumour necrosis factor alpha and interferons**. Human microvascular endothelial cells (HMVEC) were incubated with tumour necrosis factor alpha (TNF-α) in combination with IFN-α, IFN-β or IFN-γ. Results represent the mean CXCL8 and CXCL10 concentration (ng/ml) measured in the culture supernatant (three or more independent experiments).

HMVEC not only play a crucial role in leukocyte extravasation during inflammatory processes, but also form a rich source of chemokines and are targets for angiogenic chemokines (e.g. CXCL8) and antiangiogenic chemokines (e.g. CXCL10). Similar to fibroblasts, synergistic CXCL10 induction occurred between IL-1β or TNF-α and IFN-γ, whereas the cooperation between IL-1β or TNF-α and IFN-α or IFN-β was less pronounced (IFN-β) to rather weak (IFN-α) (Figures 4 and 5). HMVEC, in contrast to fibroblasts, however, required 100-fold lower amounts of IFN-γ to obtain similar levels of CXCL10 in the culture supernatant. Moreover, the cell density of the *in vitro *cultures was about fivefold lower for HMVEC compared with fibroblasts. As in fibroblasts, no synergy between IL-1β or TNF-α and IFNs was observed for CXCL8 production in HMVEC (Figures 4 and 5).

### Biochemical and biological characterisation of CXCL10 isoforms from fibroblasts

**Figure 6:**
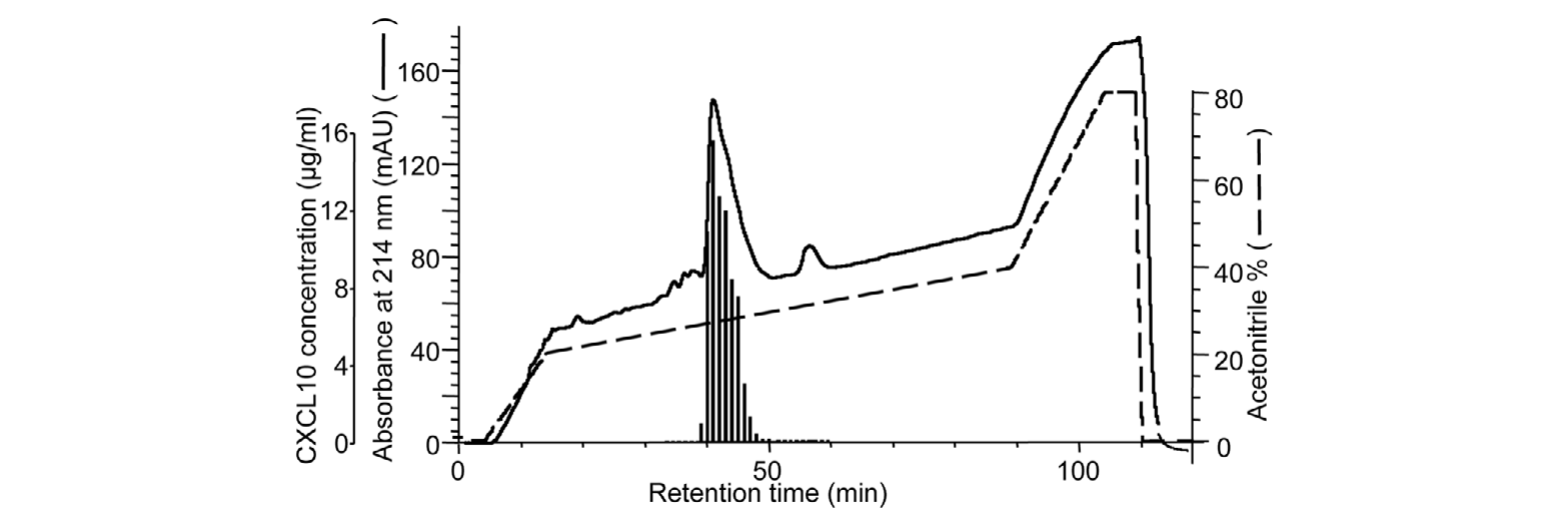
**Reverse-phase HPLC purification of fibroblast-derived CXCL10**. Semi-purified fibroblast-derived CXCL10 was subjected to C8 reverse-phase HPLC. Proteins were eluted in an acetonitrile gradient (dashed line) and UV absorbance was detected at 214 nm (solid line). CXCL10 immunoreactivity in the column fractions was detected by ELISA (histograms).

The conditioned medium from fibroblast cultures stimulated with inflammatory mediators was first concentrated by adsorption to controlled pore glass, and then chemokine fractionation was achieved upon subsequent heparin Sepharose affinity chromatography. The CXCL10 immunoreactivity eluted in a single peak between 0.7 M and 1.15 M NaCl, after the CXCL8 peak (data not shown). Further purification of CXCL10 was obtained by cation exchange chromatography. CXCL10 eluted between 0.65 M and 0.75 M NaCl from the Mono S column and was finally purified to homogeneity by C8 RP-HPLC (Figure 6). The majority of CXCL10 immunoreactivity eluted from the C8 column between 40 minutes and 46 minutes (26–29% acetonitrile).

**Figure 7:**
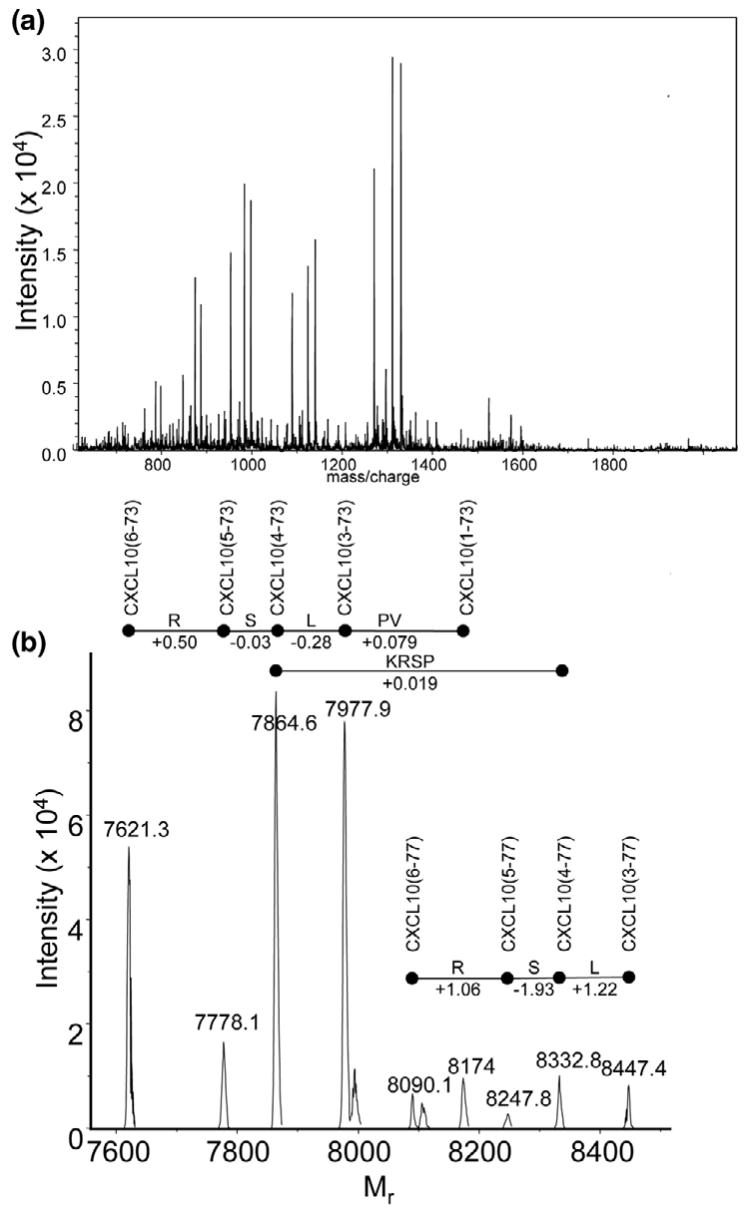
**Identification of fibroblast-derived CXCL10**. The relative molecular mass (*M*_r_) of reverse-phase-HPLC-purified CXCL10 was determined by electrospray ion trap mass spectrometry. Results show the **(a) **averaged and **(b) **averaged deconvoluted spectra of CXCL10 that eluted in between 26% and 28% acetonitrile from the C8 column (Figure 6). The amino acids cleaved off (one-letter code), explaining the differences between the CXCL10 isoforms, are indicated on top of the averaged deconvoluted spectrum. Both NH_2_-terminally truncated, COOH-terminally intact CXCL10(3–77), CXCL10(4–77), CXCL10(5–77), CXCL10(6–77), and NH_2_-terminally and COOH-terminally cleaved CXCL10(3–73), CXCL10(4–73), CXCL10(5–73) and CXCL10(6–73) were identified. The deviation between the theoretical and the experimentally determined average *M*_r _for each amino acid is indicated below the one-letter code.

Mass spectrometry revealed that at this stage CXCL10 was still heterogeneous since molecules with different *M*_r _were detected upon deconvolution of the spectra (Figure 7). The *M*_r _of all observed proteins, however, fitted with the theoretical *M*_r _of specific NH_2_-terminally truncated and/or COOH-terminally truncated forms of CXCL10. Edman degradation confirmed the existence of the different NH_2_-terminally truncated CXCL10 forms.

### Comparison of signalling activity of intact and truncated CXCL10

**Figure 8:**
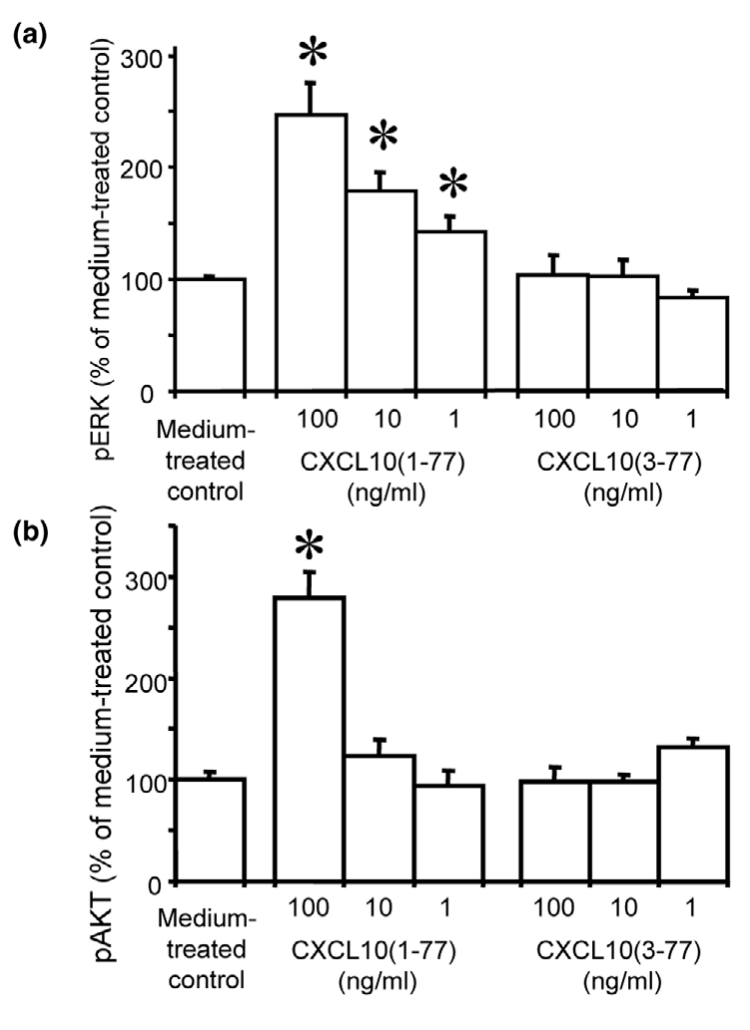
**CXCR3-dependent signalling**. Serum-starved Chinese hamster ovary CXCR3 cells were treated with Ham's F-12 medium supplemented with 0.5% foetal bovine serum (FBS) or stimulated with CXCL10 or NH_2_-terminally truncated CXCL10(3–77) at a concentration of 1, 10 or 100 ng/ml (in Ham's F-12 supplemented with 0.5% FBS). The reaction was stopped after 5 minutes and the cells were lysed. The level of extracellular signal-regulated kinase 1/2 (ERK1/2) phosphorylation or protein kinase B/Akt phosphorylation in the cell lysate was determined with specific ELISAs for phosphoERK or phosphoAkt. The mean values (*n *= 4) and standard errors are indicated. *Statistically significant differences (Mann–Whitney U test) from control (*P *< 0.05).

The two most abundant CXCL10 isoforms were missing two or three NH_2_-terminal residues. In particular, the CXCL10(3–73) isoform missing its two NH_2_-terminal residues was interesting, since this isoform can be generated *in vitro *through proteolytic cleavage of CXCL10 by soluble DPP IV (designated CD26) [22]. CHO cells transfected with CXCR3 were incubated with different concentrations of recombinant intact and CD26/DPP IV-truncated CXCL10. Intact CXCL10 at a concentration as low as 1 ng/ml was able to induce significant ERK1/2 phosphorylation in CHO/CXCR3 cells within 5 minutes (Figure 8a). Phosphorylation of Akt was obtained upon stimulation of the CHO/CXCR3 cells with 100 ng/ml intact CXCL10. In contrast, no ERK1/2 or Akt phosphorylation was observed upon treatment of CHO/CXCR3-transfected cells with CXCL10(3–77) at concentrations up to 100 ng/ml.

### Regulation of CD26/DPP IV expression and DPP IV activity in fibroblasts

**Figure 9:**
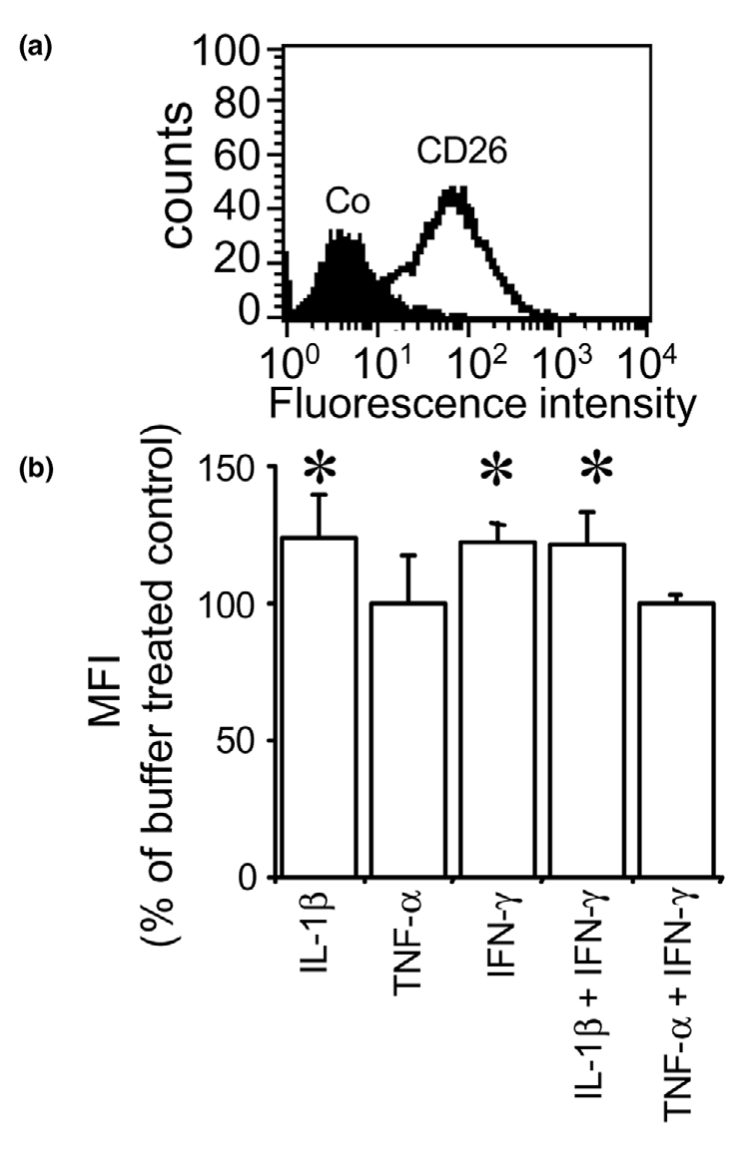
**Detection of CD26 by Fluorescence-activated cell sorting (FACS) analysis**. Expression of CD26 was detected by FACS analysis. **(a) **Expression level of CD26 on unstimulated fibroblasts. Background staining with secondary antibody only (black histograms) was compared with specific CD26-staining (open histograms). Control staining with isotype antibodies resulted in a similar histogram as with secondary antibody alone. Confluent fibroblast monolayers were left untreated (Co) or were treated with IL-1β (100 U/ml), tumour necrosis factor alpha (TNF-α) (10 ng/ml), IFN-γ (200 ng/ml) or with combinations of these cytokines. **(b) **Regulation of CD26 expression as the percentage of the relative mean fluorescence intensity (MFI) for untreated fibroblasts (± standard error of the mean). The mean MFI of four experiments is shown (except for treatment with IFN-γ alone, for which *n *= 3). Statistical analysis was performed with the Mann–Whitney U test, *P < 0.05.

**Figure 10:**
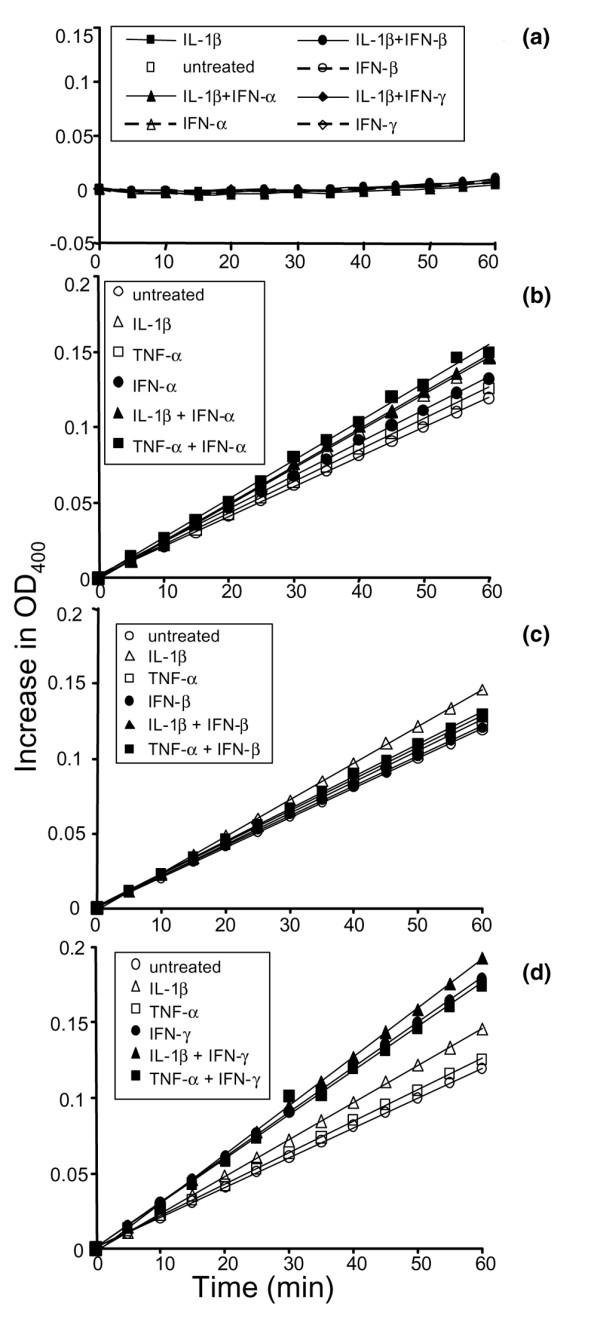
**Detection of dipeptidyl peptidase IV activity**. **(a) **Soluble dipeptidyl peptidase IV (DPP IV) activity in serum-free conditioned medium from fibroblast cultures or **(b)–(d) **the activity of DPP IV associated with fibroblast membranes was evaluated by detecting the release of *p*-nitroanilide from GlyPro-*p*-nitroanilide (increase in UV absorption at 400 nm (OD_400_) compared with culture medium or untreated cells). Fibroblasts were either left untreated or were treated with the cytokines IFN-α (1000 U/ml), IFN-β (1000 U/ml), IFN-γ (200 ng/ml), IL-1β (10 U/ml) or tumour necrosis factor alpha (TNF-α) (10 ng/ml), or combinations thereof. A significantly increased membrane-bound DPP IV activity was detected on fibroblasts treated with IFN-γ (*P *< 0.0008), with IL-1β + IFN-γ (*P *< 0.0004) or with TNF-α + IFN-γ (*P *< 0.002). Statistical analysis for DPP IV activity on cytokine-treated versus untreated fibroblasts (*n *= 4 for cytokine combinations and *n *= 8 for individual cytokines) was performed with the Student *t *test with Bonferroni correction.

The fact that fibroblasts are a cellular source of CXCL10 missing the two NH_2_-terminal residues indicates that CD26/DPP IV may be functionally expressed on these cells. In addition to CD26, the related enzyme fibroblast activation protein, is also capable of cleaving post-proline bonds and may be responsible for the observed DPP IV activity [31]. FACS analysis on fibroblast cultures, used to study CXCL10 expression, confirmed the presence of membrane-bound CD26 protein (Figure 9). Since CD26 also exists in a shed soluble form [32], DPP IV activity was analysed in fibroblast cultures as well as in the culture supernatant with a substrate conversion assay. Although membrane-bound DPP IV activity was detected on fibroblasts, there was no soluble DPP IV activity present in the culture supernatant (Figure 10a).

To investigate whether DPP IV activity (or CD26 expression) could be upregulated in fibroblasts by cytokines under similar conditions to those used to induce CXCL10, cell cultures were stimulated with IL-1β, TNF-α, IFN-α, IFN-β or IFN-γ, or mixtures thereof, in serum-free medium. Fibroblast-derived DPP IV activity was, however, not detected in the conditioned medium with the substrate conversion assay (Figure 10a) and no soluble CD26 protein was detected by ELISA (data not shown), – although CXCL10 immunoreactivity was produced as previously shown (Figure 1). Induction of fibroblasts with IL-1β or TNF-α in the presence or absence of IFN-α or IFN-β did not significantly affect membrane-bound activity of DPP IV on fibroblasts (Figure 10b,c). However, treatment of fibroblast cultures with IFN-γ alone or with IFN-γ in combination with IL-1β or TNF-α resulted in a modest but significant increase of membrane-associated DPP IV activity (Figure 10d). FACS analysis confirmed the slightly increased CD26 expression on IFN-γ-treated and IL-1β-treated fibroblasts (Figure 9b).

### Enhanced levels of CXCR3 ligands in rheumatic disorders

**Figure 11:**
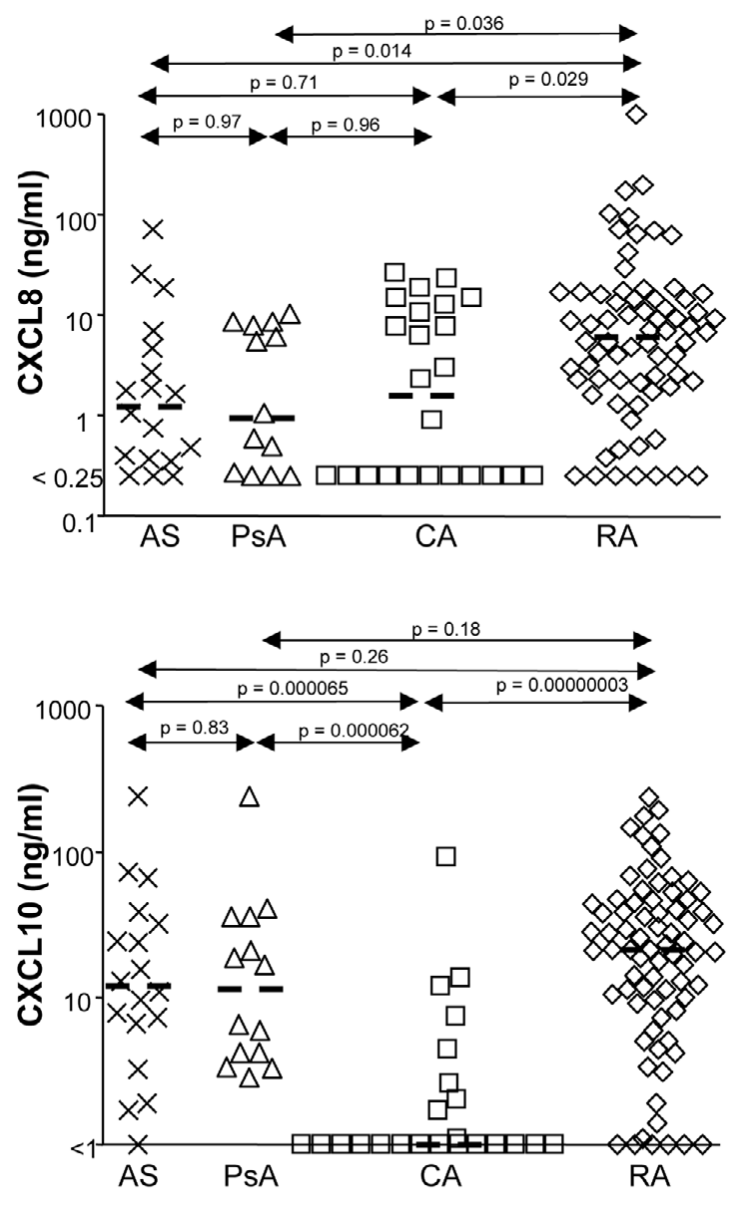
**CXCL8 and CXCL10 in synovial fluid of arthritis patients**. **(a) **CXCL10 and **(b) **CXCL8 concentrations were measured by ELISAs in synovial fluids of patients with ankylosing spondylitis (AS), psoriatic arthritis (PsA) and rheumatoid arthritis (RA), and were compared with chemokine concentrations in the metabolic arthritis patients with crystal-induced arthritis (CA). The detection limits of the ELISAs for the synovial concentrations of CXCL8 and CXCL10 are indicated on the *y *axis (logarithmic scale) and were 0.25 ng/ml and 1 ng/ml, respectively. Statistical analysis was performed with the median levels (dashed bars) using the nonparametric Mann–Whitney U test.

Synovial fluids from patients (*n *= 126) with rheumatic diseases including RA, AS, PsA and CA were analysed for their CXCL8 and CXCL10 content by specific ELISAs (Figure 11). Compared with CA patients, the median synovial CXCL10 levels were significantly enhanced in patients with RA (*P *< 10^-7^), in patients with AS (*P *< 10^-4^) and in patients with PsA (*P *< 10^-4^). No statistically significant difference in synovial fluid concentrations of CXCL10 was observed between the three types of autoimmune rheumatic disorders. The median CXCL10 concentration for the three types of autoimmune arthritis varied between 10–20 ng/ml, versus <1 ng/ml for CA. The mean level of synovial CXCL10 in the autoimmune arthritis patients was comparable with that measured in septic arthritis [11].

In contrast to CXCL10, synovial CXCL8 concentrations were only significantly (*P *< 0.05) enhanced in RA patients, and not in PsA or AS patients, in comparison with CA patients (Figure 11). This indicates that not the neutrophil chemoattractant CXCL8, but rather the Th1 lymphocyte chemoattractant CXCL10 is implicated in PsA and in AS, whereas none of the chemokines are associated with CA. No correlation was detected between CXCL8 and CXCL10 levels nor between CXCL8 or CXCL10 and serum C-reactive protein levels (data not shown).

## Discussion

IL-1β and TNF-α are potent inducers of the prototypic neutrophil chemotactic cytokine CXCL8, whereas IFN-γ is generally accepted to be the main endogenous inducer of CXCL10, which attracts and activates Th1 lymphocytes and natural killer cells [33]. Although during inflammatory conditions multiple cytokines and proteases are simultaneously produced in tissues, limited information is available on the combined effect of cytokines and proteases on chemokine production and activity in different cellular systems.

Compared with IFN-α and IFN-β, IFN-γ was the most potent stimulus of CXCL10 production in HMVEC and fibroblasts. In comparison with fibroblasts, however, HMVEC needed 100-fold lower amounts of IFN-γ to produce a comparable amount of CXCL10. Although TNF-α and IL-1β did not induce CXCL10 production in fibroblasts or HMVEC, the combined treatment of these cells with IFN-γ plus IL-1β or with IFN-γ plus TNF-α resulted in more than 10-fold increased CXCL10 protein production. Simultaneous treatment of fibroblasts or HMVEC with IFN-α or IFN-β, together with IL-1β or TNF-α resulted in a more modest synergistic increase of CXCL10 production. Cotreatment of fibroblasts with IFN-γ and IFN-α or IFN-β did not result in a significant synergistic CXCL10 production. Although TNF-α and IL-1β were reported to induce IFN-β in fibroblasts [34], IFN-β production is probably not a mediator of the observed cytokine synergy in these cells. Compared with fibroblasts, HMVEC cultures did grow to a much lower cell density. The CXCL8 and CXCL10 production, however, was much higher in HMVEC than in fibroblast cultures. The CXCL10 concentrations in the culture supernatants, therefore, despite the lower doses of IFN-γ that were used, were comparable for both cell types. The lining endothelial cells of capillaries, in direct contact with the target leukocytes in the bloodstream, are therefore an important CXCL8 and CXCL10 source. These findings are particularly interesting in view of the angiogenic and antiangiogenic activities of CXCL8 and CXCL10, respectively.

The overall chemokine activity is not only determined by its production level, but also by the interaction between chemokines and constitutive or coproduced proteases [35]. Thrombin and plasmin were reported to truncate CXCL8 by removing five NH_2_-terminal amino acids to generate a more active CXCL8 isoform, previously isolated as an important CXCL8 form from both fibroblasts and endothelial cells [29,36-38]. *In vitro*, the membrane-bound or soluble protease DPP IV or CD26 rapidly truncates CXCL10 (4 min half-life at a physiological CD26 concentration) [22]. The resulting CXCL10 isoform missing the two NH_2_-terminal amino acids lacks inflammatory activity (calcium signalling and chemotaxis through CXCR3) but retains its antiangiogenic properties [22].

Purification of CXCL10 from fibroblast cultures led to the identification of different CXCL10 isoforms by a combination of mass spectrometry and Edman degradation analysis. The two major CXCL10 isoforms that were identified were missing two or three NH_2_-terminal residues as well as missing four COOH-terminal amino acids. Activation of CXCR3 has also been associated with the ERK1/2 and protein kinase B/Akt signalling pathways, although these pathways are not required for actin polymerisation and chemotaxis [39]. CXCR3-dependent ERK1/2 or protein kinase B/Akt phosphorylation, however, is also lost upon truncation of CXCL10 by CD26 (Figure 8). The retained antiangiogenic activity of NH_2_-terminally truncated CXCL10 therefore does not seem to depend on these signalling pathways. The COOH-terminal truncation observed with the fibroblast-derived CXCL10 corresponds to the previously reported cleavage of keratinocyte-derived CXCL10 with furin, and was reported not to influence the biological activity of CXCL10 [40].

Fibroblast-derived CD26/DPP IV is likely to be responsible for the observed NH_2_-terminal truncation since CD26 expression and DPP IV activity were detected on fibroblast membranes. Although CD26/DPP IV was constitutively present on fibroblasts in an active form, IFN-γ or combinations of IFN-γ and IL-1β or TNF-α upregulated membrane-bound DPP IV activity on fibroblasts. Also, capillary endothelial cells possessed membrane-bound CD26/DPP IV (data not shown).

Chemokines and CD26/DPP IV play an important role in autoimmune diseases such as RA. CD26 protein and DPP IV activity were reported to be decreased in sera, but not in synovial fluid, from inflammatory RA patients [41]. Furthermore, antigen-induced arthritis was more severe in CD26-deficient mice [41]. As shown here, synovial CXCL10 levels were significantly increased in AS, PsA and RA compared with levels in nonautoimmune CA. Surprisingly, synovial CXCL8 concentrations were not increased in AS and PsA, and were significantly lower compared with those in RA. AS and PsA may be classified as enthesial-based arthropathies that in general have a better prognosis compared with synovial-based arthropathies such as RA [42]. Synovial CXCL8 concentrations might therefore be a useful element in the evaluation of the disease prognosis. Moreover, our data underscore the crucial role for IFN-γ, the main inducer of CXCL10, in the pathology of AS and may explain why neutrophil infiltration is less prominent in animal models of AS compared with RA [43]. Novel analytical techniques need to be developed to determine the relative amount of chemokine that is processed by proteases in synovial fluids in order to further unravel the complex interplay between cytokines, chemokines and proteases in joint inflammation.

## Conclusion

Taken together, the data described here suggest the following model. Under inflammatory conditions, primary cytokines such as TNF-α and IL-1β directly induce CXCL8 expression in tissue cells such as fibroblasts to rapidly chemoattract neutrophils. At a later stage, neutrophil influx is moderately reduced by IFNs (which are coinduced with, but partially antagonise production of, CXCL8 in fibroblasts by IL-1β and TNF-α). In contrast, IFNs synergise with IL-1β or TNF-α to produce CXCL10 and provoke a selective infiltration of activated Th1 lymphocytes and natural killer cells.

Tissue cells, however, including fibroblasts, also express chemokine degrading enzymes such as DPP IV/CD26, the expression of which is upregulated on fibroblasts in response to IFN-γ. In addition, CD26 is coexpressed with CXCR3 on the infiltrating Th1 lymphocytes and natural killer cells, and rapidly inactivates CXCL10 by NH_2_-terminal processing [22,44]. These CXCR3-expressing cells themselves therefore also contribute to CXCL10 degradation, providing a negative feedback loop that prevents further infiltration of activated Th1 lymphocytes and natural killer cells to the site of inflammation.

In conclusion, a network of interactions between cytokines, chemokines and proteases controls the recruitment of leukocytes. Blockage of a single cytokine *in vivo *(e.g. TNF-α or IL-1β) in autoimmune arthritis by antibodies or soluble receptor antagonists does prevent its synergistic interaction with other cytokines to induce chemokines and other inflammatory mediators, and probably explains the significant medical benefit of such selective therapy [24,45].

## Abbreviations

AS = ankylosing spondylitis; CA = crystal-induced arthritis; CHO = Chinese hamster ovary; CXCL = CXC ligand; CXCR = CXC receptor; DPP IV = dipeptidyl peptidase IV; ELISA = enzyme-linked immunosorbent assay; ERK = extracellular signal-regulated kinase; FACS = Fluorescence-activated cell sorting; FBS = foetal bovine serum; HMVEC = human microvascular endothelial cells; HPLC = high-performance liquid chromatography; IL = interleukin; IFN = interferon; LPS = lipopolysaccharide; MEM = Eagle's minimal essential medium; *M*_r _= relative molecular mass; OD_400 _= UV absorption at 400 nm; PBS = phosphate-buffered saline; PsA = psoriatic arthritis; RA = rheumatoid arthritis; RP = reverse phase; Th1 = T helper type 1; TNF-α, tumour necrosis factor alpha.

## Competing interests

The authors declare that they have no competing interests.

## Authors' contributions

PP, SS, TL, MG, ES, RC and IR were responsible for cell cultures, performed the immunoassays, participated in the purification and identification of the natural proteins, performed signal transduction assays and/or carried out the FACS analysis. MP prepared the stable CXCR3 transfectants. JB detected CD26 activity and BG collected patient samples. PP, GO and JVD participated in the design of the study and wrote the manuscript. SS, TL, MG, ES, MP, BG and JB revised the manuscript. All authors read and approved the manuscript.
